# Supplementary material for: The Heterogeneous Impact of Prediagnostic Folate Intake for Fluorouracil-Containing Induction Chemotherapy for Head and Neck Cancer
Source: Cancers (Basel). 2023 Oct 26;15(21):5150. doi: 10.3390/cancers15215150 (PMC10650771; doi:10.3390/cancers15215150)
Supplement: Supplementary file 1 [file cancers-15-05150-s001.zip › cancers-2629954-Figure S1.pdf]

Figure S1

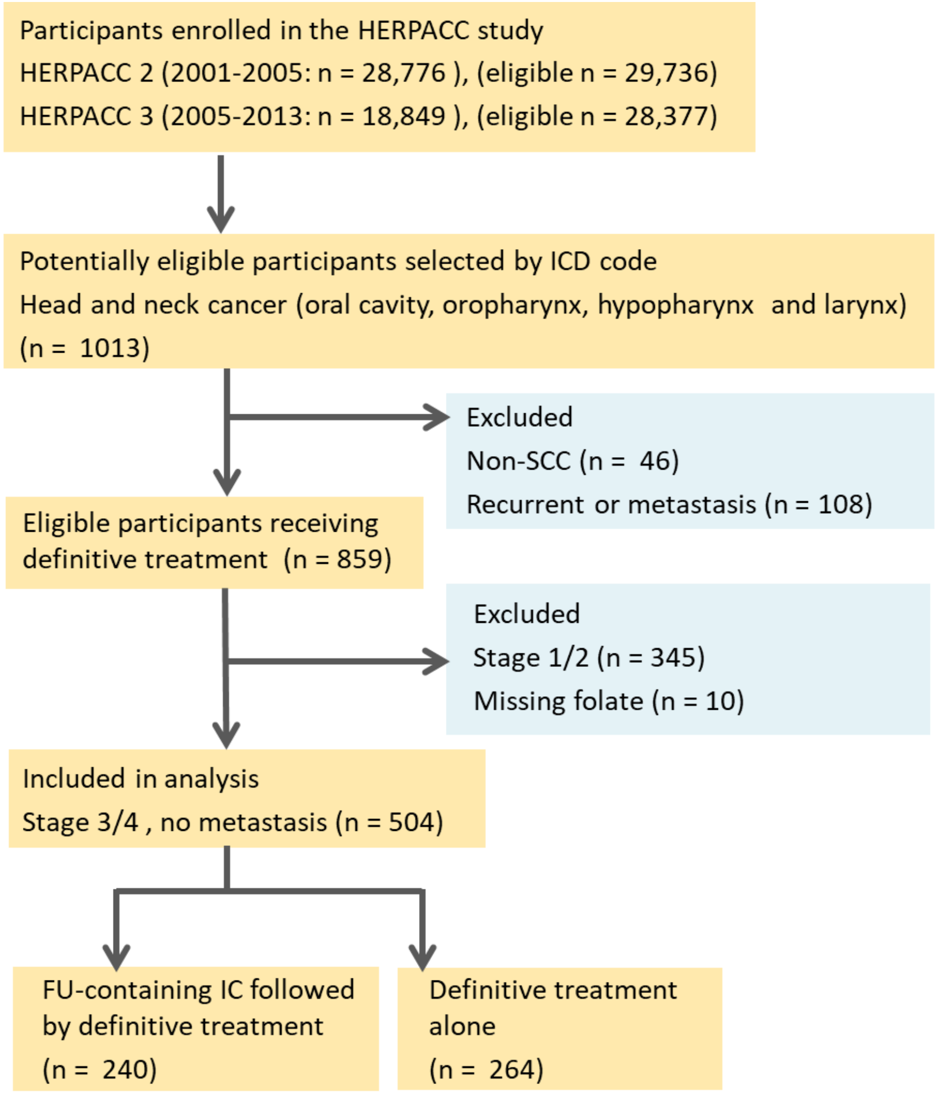

Figure S1. Flow diagram of the study design showing exclusion criteria for patients with locally advanced head and neck cancer treated by either FU-containing induction chemotherapy (IC) followed by definitive treatment or definitive treatment alone. 108 patients were excluded due to recurrent disease or metastasis, 46 with a diagnosis of not squamous cell carcinoma (SCC), 345 in stage 1-2, and 10 with missing data on folate intake.
