# Supplementary material for: The Heterogeneous Impact of Prediagnostic Folate Intake for Fluorouracil-Containing Induction Chemotherapy for Head and Neck Cancer
Source: Cancers (Basel). 2023 Oct 26;15(21):5150. doi: 10.3390/cancers15215150 (PMC10650771; doi:10.3390/cancers15215150)
Supplement: Supplementary file 1 [file cancers-15-05150-s001.zip › cancers-2629954-Figure S2.pdf]

Figure S 2

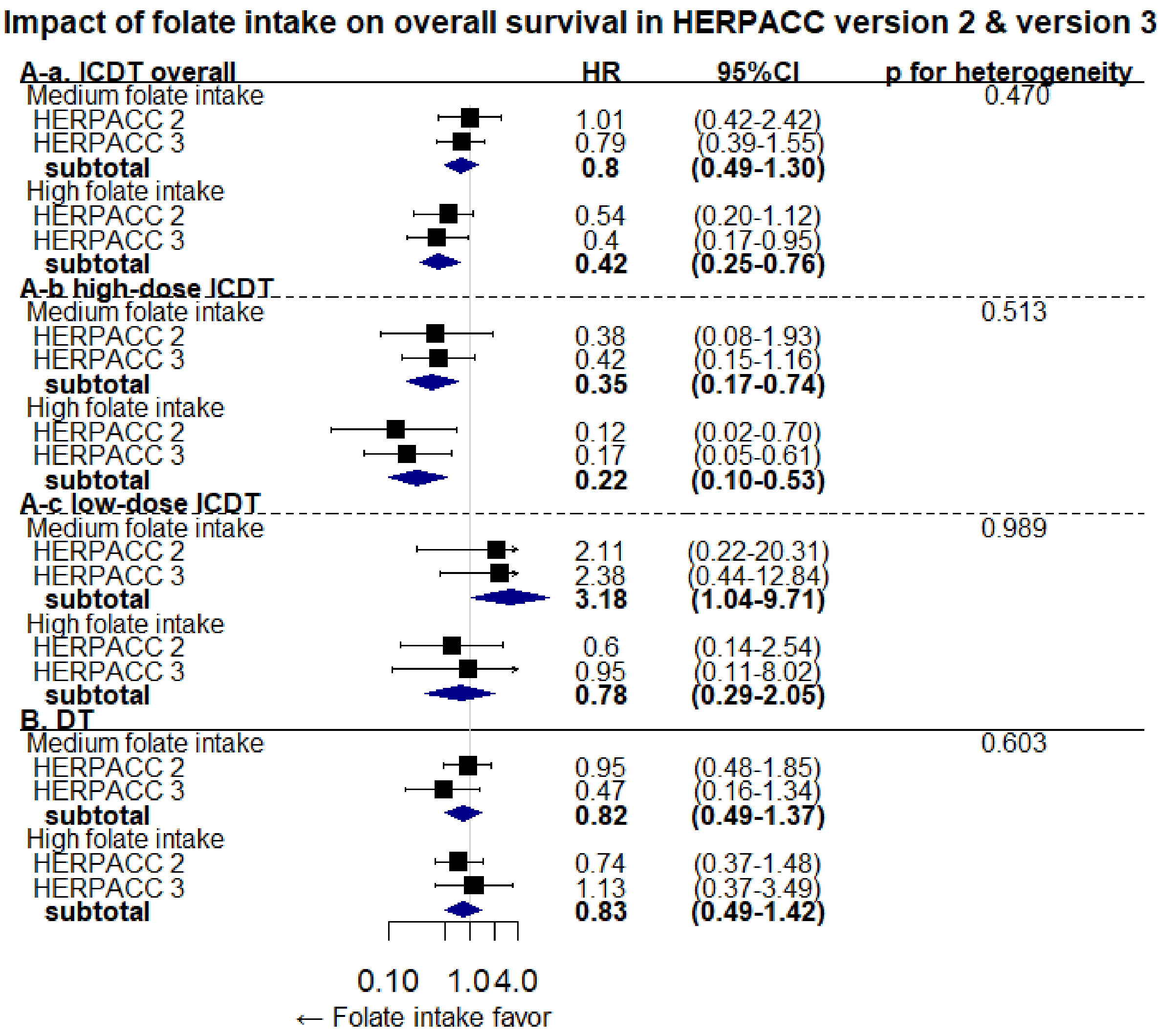

Figure S2. Impact of folate intake on OS stratified by study period. (A-a) Among patients treated by FU-containing IC followed by definitive treatment overall, an association between folate intake and survival was consistently observed in both HERPACC versions 2 and 3. Even after stratification by cumulative dose of FU, both among high- (A-b) and low-dose IC (A-c), the association between folate intake and survival was similar between HERPACC version 2 and version 3. Regarding definitive treatment alone (B), although there was a difference in survival impact of folate intake between study periods, heterogeneity was not significant. ($p = 0.603$).

p for heterogeneity: heterogeneity of survival impact of folate intake between HERPACC version 2 and HERPACC version 3.
