# Supplementary material for: The Heterogeneous Impact of Prediagnostic Folate Intake for Fluorouracil-Containing Induction Chemotherapy for Head and Neck Cancer
Source: Cancers (Basel). 2023 Oct 26;15(21):5150. doi: 10.3390/cancers15215150 (PMC10650771; doi:10.3390/cancers15215150)
Supplement: Supplementary file 1 [file cancers-15-05150-s001.zip › cancers-2629954-Figure S3.pdf]

Figure S3.

Stratification by clinical covariates

A	Event / total					
**Covariates**	**low/medium**	**high**	**HR**	**95% CI**	**P interaction**
Sex male	65/141	21/69	0.50	(0.29-0.84)	0.809
Sex female	7/18	3/12	NE		
Age <60	32/83	3/16	0.60	(0.24-1.52)	0.317
Age ≥60	40/76	16/55	0.45	(0.23-0.85)	
Oral cavity	20/39	5/13	0.26	(0.06-1.14)	0.538
Oropharynx	16/48	8/26	0.41	(0.16-1.04)	
Hypopharynx	32/59	11/26	0.69	(0.28-1.75)	
Larynx	4/13	0/5	NE		
Stage 3	15/38	2/15	0.01	(0.00-0.30)	0.107
Stage 4	57/121	22/66	0.54	(0.32-0.93)	
T 1-2	16/56	14/44	1.34	(0.54-3.33)	0.026
T 3-4	56/103	10/37	0.32	(0.15-0.66)	
N 0-1	21/55	4/21	0.16	(0.04-0.61)	0.246
N 2-3	51/104	20/60	0.47	(0.28-0.79)	
Drink non-light	23/58	5/27	0.27	(0.08-0.79)	0.135
Drink mod-heavy	48/100	19/54	0.63	(0.34 -1.18)	
Smoke non-light	18/42	7/25	0.18	(0.04-0.77)	0.569
Smoke mod-heavy	48/111	17/53	0.63	(0.35-1.13)	

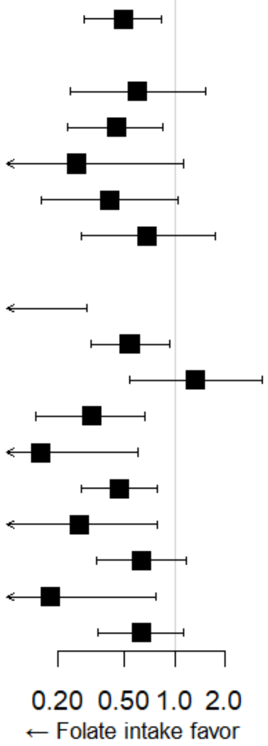

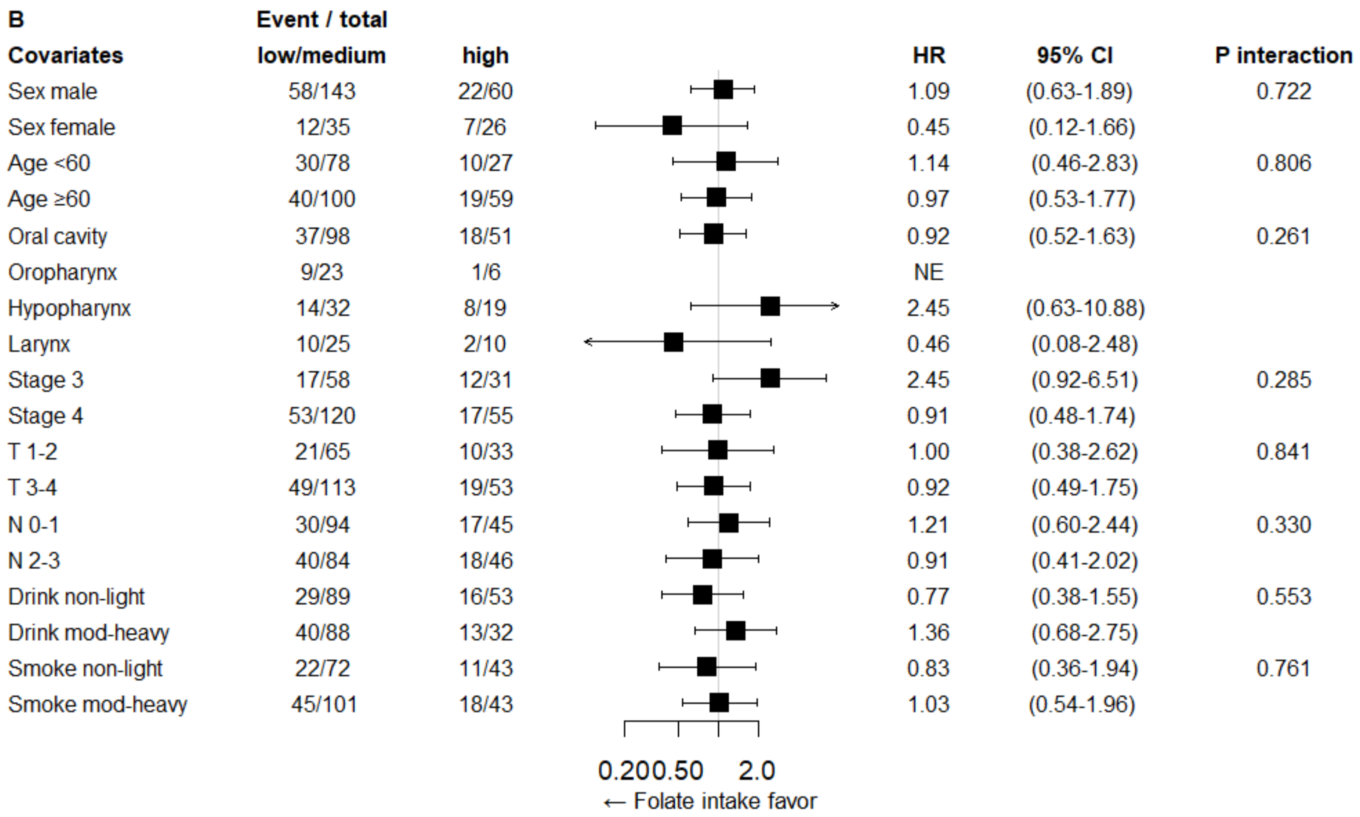

Figure S3. Hazard ratio for death with high folate intake compared to low-medium folate intake stratified by clinical covariates. (A) Among patients treated by FU-containing IC followed by definitive therapy, high folate intake tended to be associated with improved prognosis in almost all subgroups other than those with T1-2 classification. (B) Among patients treated by definitive therapy alone, high folate intake was less likely to be associated with improved prognosis in almost all subgroups, other than those with female and laryngeal cancer. Hazard ratios was adjusted by sex, age, performance status, smoking, alcohol consumption, primary site, therapy, UICC T classification, UICC N classification, energy, and vitamin supplementation.
