# Supplementary material for: The Heterogeneous Impact of Prediagnostic Folate Intake for Fluorouracil-Containing Induction Chemotherapy for Head and Neck Cancer
Source: Cancers (Basel). 2023 Oct 26;15(21):5150. doi: 10.3390/cancers15215150 (PMC10650771; doi:10.3390/cancers15215150)
Supplement: Supplementary file 1 [file cancers-15-05150-s001.zip › cancers-2629954-Table S1.pdf]

Table S1. Characteristics of patients at baseline by study period

	Total		HERPACC2 (2001-2005)		HERPACC3 (2005-20013)		
	N = 504	(%)	N = 248	(%)	N = 256	(%)	p-value†
Sex							0.959
Male	413	(82)	203	(88)	210	(82)	
Female	91	(18)	45	(13)	46	(18)	
Age (years)							0.200
<65	214	(42)	109	(45)	105	(41)	
≥65	290	(58)	139	(51)	151	(59)	
ECOG PS							0.505
0	293	(58)	111	(64)	182	(71)	
1	191	(38)	122	(34)	69	(27)	
2	20	(4)	15	(2)	5	(2)	
Primary site							0.028
Oral cavity	201	(40)	103	(22)	98	(38)	
Oropharynx	114	(23)	51	(35)	63	(25)	
Hypopharynx	136	(27)	59	(35)	77	(30)	
Larynx	53	(11)	35	(8)	18	(7)	
UICC stage							0.016
3	142	(28)	82	(22)	60	(23)	
4	362	(72)	166	(78)	169	(66)	
UICC T classification							0.177
1	33	(7)	12	(5)	21	(8)	
2	165	(33)	82	(33)	83	(32)	
3	153	(30)	86	(35)	67	(26)	
4	152	(30)	68	(27)	84	(33)	
X	1	(0)	0	(0)	1	(0)	

UICC N classification							0.719
0	104	(21)	50	(20)	54	(21)	
1	111	(22)	60	(24)	51	(20)	
2	268	(53)	128	(52)	140	(55)	
3	21	(4)	10	(4)	11	(4)	
Definitive treatment							0.013
Surgery	223	(44)	77	(31)	146	(57)	
Radiotherapy	281	(56)	171	(69)	110	(43)	
Presence or absence of FU-containing IC							0.062
Definitive treatment alone	264	(52)	141	(57)	123	(48)	
FU-containing IC followed by definitive treatment	240	(48)	107	(43)	133	(52)	
Cumulative dose of FU in terms of IC‡							0.199
High-IC	152	(30)	63	(25)	89	(35)	
Low-IC	88	(17)	44	(18)	44	(17)	
IC regimen							0.360
5-FU + CDDP (tri- weekly FP)	196	(39)	90	(36)	106	(41)	
5-FU + CDDP (weekly FP)	32	(6)	15	(6)	17	(7)	
5-FU + CBDCA	5	(1)	1	(0)	4	(2)	
5-FU + NDP	2	(0)	1	(0)	1	(0)	
5-FU + CDDP + DTX	4	(1)	0	(0)	4	(2)	
S-1 (daily) + CDDP	1	(0)	0	(0)	1	(0)	
Cumulative smoking							0.457
Non-smoker	111	(22)	51	(21)	60	(23)	
Light (< 20PY)	71	(14)	34	(14)	37	(14)	
Moderate (20PY to < 30PY)	127	(25)	66	(27)	61	(24)	
Heavy (≥ 30PY)	181	(36)	93	(38)	88	(34)	
Unknown	14	(3)	4	(2)	10	(4)	
Alcohol consumption							0.010

Non-drinker	115	(23)	53	(21)	62	(24)	
Light	112	(22)	65	(26)	47	(18)	
Moderate	107	(21)	59	(24)	48	(19)	
Heavy	167	(33)	68	(27)	99	(39)	
Unknown	3	(1)	3	(1)	0	(0)	
Continuous dietary folate intake (median ±SD, µg/day)			268 (±110)		339 (±126)		< 0.001¶
Folate intake ‡							0.746
Low	169	(34)	83	(33)	86	(34)	
Medium	168	(33)	83	(33)	85	(33)	
High	167	(33)	82	(33)	85	(33)	
Vitamin supplementation							0.094
No	423	(84)	205	(83)	218	(85)	
Yes	69	(14)	33	(13)	36	(14)	
Unknown	12	(2)	2	(1)	10	(4)	

ECOG PS, Eastern Cooperative Oncology Group performance status; UICC, International Union Against Cancer.

§ High: Cumulative dose of FU during IC was more than 8000 mg/m^2, equivalent to 2 cycles of FP 3 weekly. Low: Cumulative dose of FU during IC was less than 8000 mg/m^2 during IC.

† Chi square test or Fisher exact test ¶ Student's t-test
