# Supplementary material for: The Heterogeneous Impact of Prediagnostic Folate Intake for Fluorouracil-Containing Induction Chemotherapy for Head and Neck Cancer
Source: Cancers (Basel). 2023 Oct 26;15(21):5150. doi: 10.3390/cancers15215150 (PMC10650771; doi:10.3390/cancers15215150)
Supplement: Supplementary file 1 [file cancers-15-05150-s001.zip › cancers-2629954-Table S2.pdf]

Table S2. Regimen of induction chemotherapy

Regimen of induction chemotherapy	Schedule	Total		High cumulative dose of FU during IC term †		Low cumulative dose of FU during IC term †	
		N = 240	(%)	N = 152	(%)	N = 88	(%)
FU + CDDP (tri- weekly FP)	FU 800mg/m^2 day 1-5 + CDDP 80/m^2 day 6, every 3 weeks	196	(82)	136	(90)	60	(68)
FU + CDDP (weekly FP)	FU 800mg/ m^2 day 1-2 + CDDP 25/m^2 day 1, every 1 week	32	(13)	7	(5)	25	(28)
FU + CBDCA	FU 800mg/ m^2 day 1-5 + CBDCA AUC 5/m^2 day 5, every 3 weeks	5	(2)	3	(2)	2	(2)
FU + NDP	FU 800mg/ m^2 day 1-5 + NDP AUC 5/m^2 day 5, every 3 weeks	2	(1)	1	(1)	1	(1)
FU + CDDP + DTX	FU 600mg/ m^2 day 1-5 + CDDP 80/m^2 day 1 + DTX 60mg/m^2 day 1, every 3 weeks	4	(2)	4	(3)	0	(0.0)
S-1 + CDDP	S-1 80mg/m^2 daily 2 week + CDDP 80mg/m^2 day 1, every 3 weeks	1	(0)	1	(1)	0	(0.0)

CDDP, cisplatin; NDP, nedaplatin; CBDCA, carboplatin; DTX, docetaxel; FU, fluorouracil

† High: Cumulative dose of FU during IC was more than 8000 mg/m^2, equivalent to 2 cycles of FU + CDDP (3 weeks). Low: Cumulative dose of FU during IC was less than 8000 mg/m^2 during IC.

Formatted: German (Germany)

Formatted: German (Germany)
