# Supplementary material for: The Heterogeneous Impact of Prediagnostic Folate Intake for Fluorouracil-Containing Induction Chemotherapy for Head and Neck Cancer
Source: Cancers (Basel). 2023 Oct 26;15(21):5150. doi: 10.3390/cancers15215150 (PMC10650771; doi:10.3390/cancers15215150)
Supplement: Supplementary file 1 [file cancers-15-05150-s001.zip › cancers-2629954-Table S3.pdf]

Table S3 Impact of folate intake on overall survival by landmark analysis (landmark time =2 months)

Folate intake	FU-based IC followed by definitive treatment						p for interaction	Definitive treatment alone (DT)					
	Crude			Adjusted[‡]				Crude			Adjusted[‡]		
	HR	(95%CI)	p-value	HR	(95%CI)	p-value		HR	(95%CI)	p-value	HR	(95%CI)	p-value
Overall							0.196 [§]	1 (reference)			1 (reference)		
Low	1 (reference)			1 (reference)				0.87	(0.54 -1.40)	0.563	0.87	(0.52 -1.45)	0.594
Medium	0.87	(0.55 -1.40)	0.574	0.77	(0.47 -1.27)	0.262		0.77	(0.47 -1.27)	0.305	0.94	(0.55 -1.60)	0.806
High	0.51	(0.31 -0.86)	0.012	0.43	(0.24 -0.75)	0.002		trend p=0.299			trend p=0.789		
	trend p=0.017			trend p=0.003									
High cumulative dose of FU during IC term in IC-DT (N = 152)[§§]							0.027 [¶]						
Low	1 (reference)			1 (reference)									
Medium	0.54	(0.29 -1.00)	0.052	0.36	(0.17 -0.75)	0.007							
High	0.29	(0.13 -0.62)	0.002	0.23	(0.10 -0.55)	0.001							
	trend p=0.001			trend p=<0.001									
Low cumulative dose of FU during IC term (N = 88)[§§]							0.045[†]						
Low	1 (reference)			1 (reference)									
Medium	1.99	(0.92 -4.29)	0.081	3.45	(1.08 -11.00)	0.036							
High	0.97	(0.45 -2.12)	0.941	0.85	(0.31 -2.36)	0.762							
	trend p=0.811			trend p= 0.165									

‡ adjusted by sex, age, performance status, smoking, alcohol consumption, primary site, definitive therapy, UICC T classification, UICC N classification, energy, supplement use

§ interaction between FU-containing IC overall and definitive treatment alone, ¶ interaction between high cumulative dose of FU and definitive treatment alone. † interaction between low cumulative dose of FU and definitive treatment alone

§§ High: Cumulative dose of FU during IC terms was more than 8000 mg/m^2, equivalent to 2 cycles. Low: cumulative dose of FU during IC terms was less than 8000 mg/m^2 in terms of IC.
