# Supplementary material for: The Heterogeneous Impact of Prediagnostic Folate Intake for Fluorouracil-Containing Induction Chemotherapy for Head and Neck Cancer
Source: Cancers (Basel). 2023 Oct 26;15(21):5150. doi: 10.3390/cancers15215150 (PMC10650771; doi:10.3390/cancers15215150)
Supplement: Supplementary file 1 [file cancers-15-05150-s001.zip › cancers-2629954-Table S4.pdf]

Table S4 Impact of folate intake on recurrence-free survival and distant metastasis-free survival

	**FU-based IC followed by definitive treatment (IC-DT) N = 240**						**Definitive treatment alone (DT) N = 264**						
Folate intake†	N (%)	5-year OS (%) (95%CI)	Crude HR (95%CI)	p-value	Adjusted‡HR (95%CI)	p-value	N (%)	5-year OS (%) (95%CI)	Crude HR (95%CI)	p-value	Adjusted‡HR (95%CI)	p-value	p for interaction
**Recurrence-free survival**													
**Overall**													0.631§
Low	83 (35)	41.2 (30.1-52.2)	1 (reference)		1 (reference)		86 (33)	40.4 (28.4-52.3)	1 (reference)		1 (reference)		
Medium	76 (32)	37.0 (26.2-48.5)	1.13 (0.76-1.69)	0.547	1.08 (0.71-1.62)	0.731	92 (35)	48.3 (37.0-58.8)	0.87 (0.59-1.31)	0.524	0.92 (0.60-1.42)	0.677	
High	81 (34)	44.4 (33.0-55.0)	0.90 (0.60-1.36)	0.624	0.88 (0.58-1.36)	0.576	86 (33)	57.0 (45.0-67.3)	0.72 (0.46-1.10)	0.129	0.82 (0.51-1.30)	0.495	
			trend p=0.600		trend p=0.580				trend p=0.129		trend p=0.395		
**Stratification by cumulative dose of FU during IC terms**													
High cumulative dose of FU during IC term in FU-containing IC (N = 152)§§													0.912¶
Low	53 (35)	38.3 (24.6-52.0)	1 (reference)		1 (reference)								
Medium	54 (36)	42.7 (28.5-56.1)	0.87 (0.53-1.43)	0.592	0.69 (0.40-1.20)	0.188							
High	45 (30)	44.9 (29.6-59.1)	0.79 (0.47-1.34)	0.391	0.79 (0.45-1.39)	0.416							
			trend p=0.390		trend p=0.003								
Low cumulative dose of FU during IC term (N = 88)§§													0.897
Low	30 (34)	46.8 (27.6-64.0)	1 (reference)		1 (reference)								
Medium	22 (25)	22.7 (8.3-41.5)	2.00 (0.99-4.02)	0.053	3.17 (1.27-7.89)	0.013							
High	36 (41)	43.9 (27.4-59.2)	1.13 (0.58-2.18)	0.726	1.13 (0.49-2.58)	0.771							
			trend p=0.842		trend p=0.990								
**Stratification by definitive treatment**													
Surgery	N = 97						N = 126						0.224§
Low	27 (28)	44.3(34.9-71.7)	1 (reference)		1 (reference)		35 (28)	38.3(19.7-56.8)	1 (reference)		1 (reference)		
Medium	37 (38)	34.3(19.6-49.7)	1.58 (0.82-3.03)	0.166	1.94 (0.92-4.11)	0.082	51 (40)	50.2(34.1-64.3)	0.83 (0.46-1.51)	0.557	1.00 (0.52-1.90)	0.992	
High	33 (34)	34.5(18.6-51.0)	1.27 (0.65-2.50)	0.477	1.04 (0.49-2.18)	0.919	40 (32)	61.1(43.8-74.5)	0.63 (0.32-1.24)	0.188	0.69 (0.32-1.49)	0.342	

			trend p=0.558		trend p=0.849				trend p=0.188		trend p=0.371		
Radiotherapy	N =143						N = 138						0.897
Low	56(39)	40.1(26.1-52.7)	1 (reference)		1 (reference)		51(37)	41.7(26.0-56.6)	1 (reference)		1 (reference)		
Medium	39(27)	39.7(23.4-55.5)	0.84 (0.50-1.46)	0.553	0.70 (0.38-1.26)	0.232	41(30)	47.9(31.9-62.3)	0.93 (0.52-1.62)	0.806	0.92 (0.50-1.70)	0.790	
High	48(34)	51.7(36.6-64.7)	0.71 (0.41-1.20)	0.205	0.87 (0.48-1.57)	0.643	46(33)	53.0(36.0-67.6)	0.78 (0.44-1.38)	0.397	0.77 (0.41-1.44)	0.417	
			trend p=0.205		trend p=0.574				trend p=0.400		trend p=0.421		
**Distant metastasis-free survival**													
**Overall**													0.309§
Low	80(35)	50.1 (38.2-60.8)	1 (reference)		1 (reference)		89(32)	54.9 (42.2-65.9)	1 (reference)		1 (reference)		
Medium	74(32)	49.6 (37.6-60.5)	1.03 (0.67-1.60)	0.889	0.92 (0.58-1.52)	0.729	94(34)	56.2 (44.8-66.1)	0.99 (0.63-1.55)	0.970	0.99 (0.61-1.60)	0.974	
High	76(33)	69.0 (57.3-78.1)	0.52 (0.32-0.86)	0.011	0.41 (0.25-0.74)	0.001	91(33)	67.3 (55.6-76.6)	0.77 (0.48-1.24)	0.283	0.92 (0.55-1.55)	0.756	
			trend p=0.011		trend p=0.001			80(35)	trend p=0.285		trend p=0.761		
**Stratification by cumulative dose of FU during IC terms**													
High cumulative dose of FU during IC term (N = 152)§§													0.039¶
Low	53(35)	47.2 (32.3-60.8)	1 (reference)		1 (reference)								
Medium	54(36)	59.5 (44.7-60.5)	0.73 (0.42-1.29)	0.282	0.56 (0.29-1.11)	0.098							
High	45(30)	78.4 (62.2-78.1)	0.32 (0.15-0.68)	0.003	0.28 (0.12-0.61)	0.002							
			trend p=0.002		trend p=0.001								
Low cumulative dose of FU during IC term (N = 88)§§													
Low	30(34)	54.6 (34.8-70.7)	1 (reference)		1 (reference)								0.843†
Medium	22(25)	27.3 (11.1-46.4)	1.95 (0.95-3.98)	0.069	3.42 (1.19-9.81)	0.022							
High	36(41)	57.1 (39.0-71.6)	0.87 (0.42-1.81)	0.710	0.72 (0.28-1.81)	0.484							
			trend p=0.600		trend p=0.212								
**Stratification by definitive treatment**													
Surgery	N = 97						N = 126						0.214§
Low	27(28)	52.8 (31.6-70.2)	1 (reference)		1 (reference)		35(28)	60.1 (40.3-75.2)	1 (reference)		1 (reference)		
Medium	37(38)	41.8 (25.6-57.1)	1.44 (0.71-2.90)	0.302	0.97 (0.44-2.17)	0.949	51(40)	59.0 (43.7-71.4)	0.95 (0.48-1.85)	0.881	1.02 (0.47-2.19)	0.970	
High	33(34)	64.2 (44.3-78.6)	0.64 (0.28-1.47)	0.3	0.33 (0.13-0.84)	0.019	40(32)	70.7 (53.3-82.7)	0.59 (0.27-1.29)	0.19	0.84 (0.34-2.11)	0.712	

			trend p= 0.262		trend p=0.013				trend p= 0.189		trend p=0.215		
Radiotherapy	N =143						N = 138						
Low	56(39)	48.5 (34.0-61.4)	1 (reference)		1 (reference)		51(37)	52.0 (36.0-67.0)	1 (reference)		1 (reference)		0.843[§]
Medium	39(27)	57.6 (40.2-71.6)	0.71 (0.38-1.32)	0.289	0.72 (0.36-1.43)	0.342	41(30)	57.3 (40.4-71.0)	0.99 (0.53-1.82)	0.974	0.75 (0.37-1.51)	0.415	
High	48(34)	72.4 (57.2-83.0)	0.44 (0.23-0.85)	0.015	0.49 (0.24-1.01)	0.053	46(33)	63.3 (46.0-76.4)	0.81 (0.42-1.51)	0.514	0.64 (0.31-1.30)	0.214	
			trend p=0.014		trend p=0.050				trend p= 0.520		trend p=0.215		

‡ adjusted by sex, age, performance status, smoking, alcohol consumption, primary site, definitive therapy, UICC T classification, UICC N classification, energy, supplement use

§ interaction between FU-based IC and definitive treatment alone, ¶ interaction between high, cumulative dose of FU and definitive treatment alone

† interaction between low cumulative dose of FU and definitive treatment alone

§§ High: Cumulative dose of FU during IC terms was more than 8000 mg/m^2, equivalent to 2 cycles. Low: cumulative dose of FU during IC terms was less than 8000 mg/m^2 in terms of IC.
