# Supplementary material for: The Heterogeneous Impact of Prediagnostic Folate Intake for Fluorouracil-Containing Induction Chemotherapy for Head and Neck Cancer
Source: Cancers (Basel). 2023 Oct 26;15(21):5150. doi: 10.3390/cancers15215150 (PMC10650771; doi:10.3390/cancers15215150)
Supplement: Supplementary file 1 [file cancers-15-05150-s001.zip › cancers-2629954-Table S5.pdf]

Table S5. Multivariate analysis for nutrients other than folate

	Median		FU-containing IC followed by definitive treatment			Definitive treatment alone		
Nutrient	HERPACC 2	HERPACC 3	Adjusted HR†	(95%CI)	p-value	Adjusted HR†	(95%CI)	p-value
Carbohydrate (g)	223.47	252.98	0.98	(0.64 -1.51)	0.930	1.17	(0.68 -2.01)	0.561
Protein (g)	45.94	54.67	1.04	(0.67 -1.59)	0.870	0.97	(0.58 -1.62)	0.895
Fat (g)	33.37	40.54	1.10	(0.71 -1.71)	0.680	0.86	(0.55 -1.33)	0.493
Sodium (mg)	1611.00	1964.22	1.14	(0.74 -1.75)	0.566	1.12	(0.70 -1.79)	0.636
Potassium (mg)	1811.79	2224.07	0.81	(0.49 -1.32)	0.392	0.94	(0.57 -1.56)	0.808
Carotene (ng)	2132.16	2639.43	0.57	(0.33 -0.99)	0.045	0.90	(0.54 -1.51)	0.700
Calcium (mg)	426.13	521.46	1.08	(0.70 -1.66)	0.732	0.84	(0.53 -1.31)	0.439
Fe (mg)	4.62	5.73	0.76	(0.45 -1.28)	0.301	0.89	(0.49 -1.61)	0.699
Retinoid (mg)	785.38	803.88	1.37	(0.88 -2.13)	0.162	0.76	(0.46 -1.27)	0.294
Vitamin D (μg)	5.55	5.61	0.91	(0.58 -1.43)	0.679	0.80	(0.50 -1.29)	0.355
Vitamin E (μg)	4.91	6.21	0.90	(0.57 -1.43)	0.656	1.17	(0.72 -1.90)	0.524
Vitamin B1 (μg)	1.07	0.97	0.90	(0.58 -1.40)	0.636	0.85	(0.55 -1.33)	0.484
Vitamin B2 (μg)	0.96	0.98	1.01	(0.64 -1.58)	0.974	0.66	(0.40 -1.09)	0.104
Vitamin C (μg)	69.42	86.38	0.55	(0.27 -1.13)	0.102	1.08	(0.60 -1.95)	0.801
SUFA (g)	7.57	8.99	0.89	(0.57 -1.37)	0.583	0.87	(0.56 -1.33)	0.515
MUFA (g)	11.25	13.80	0.92	(0.57 -1.47)	0.716	0.99	(0.64 -1.55)	0.976
PUFA (g)	9.19	11.10	0.77	(0.49 -1.22)	0.261	0.88	(0.55 -1.39)	0.572
Cholesterol (g)	225.62	255.70	0.86	(0.55 -1.34)	0.510	0.84	(0.54 -1.32)	0.456
SDF (g)	0.97	1.17	0.94	(0.59 -1.52)	0.810	0.98	(0.61 -1.60)	0.949
IDF (g)	4.17	5.44	1.01	(0.62 -1.64)	0.963	1.16	(0.68 -1.97)	0.581
TDF (g)	6.19	8.33	1.09	(0.67 -1.75)	0.735	1.29	(0.76 -2.21)	0.344
n-3 PUFA (mg)	2132.02	2406.94	0.89	(0.56 -1.42)	0.636	0.84	(0.54 -1.32)	0.453
n-6 PUFA (mg)	9484.07	10879.74	0.82	(0.52 -1.29)	0.387	1.05	(0.67 -1.64)	0.840

n-3 HUFA (mg)	778.52	782.13	0.93	(0.59	-1.47)	0.750	0.72	(0.45 -1.15)	0.170

† adjusted by sex, age, PS, smoking, drinking, primary site, therapy, T stage, N stage, energy, folate intake, and vitamin supplementation

HR: hazard ratio for death of over median of nutrition compared to under median of nutrition. Over median and under median were divided by study period separately. Abbreviations: SUFA, saturated fatty acid; MUFA, monounsaturated fatty acid; PUFA, poly-unsaturated fatty acid; SDF, soluble dietary fiber; IDF, insoluble dietary fiber; n-3 PUFA, omega-3 poly-unsaturated fatty acid; n-6 PUFA, omega-6 poly-unsaturated fatty acid; HUFA, omega-3 highly unsaturated fatty acid
